# Influence of Diet and Gender on Plasma DPP4 Activity and GLP-1 in Patients with Metabolic Syndrome: An Experimental Pilot Study

**DOI:** 10.3390/molecules23071564

**Published:** 2018-06-28

**Authors:** Francisco Tomás Pérez-Durillo, Ana Belén Segarra, Ana Belén Villarejo, Manuel Ramírez-Sánchez, Isabel Prieto

**Affiliations:** 1 Nutrition and Neuroendocrinology Research Group, Department of Health Sciences, University of Jaén, 23071 Jaén, Spain; t_perez@yahoo.com (F.T.P.-D.); asegarra@ujaen.es (A.B.S.); ab_villa@hotmail.com (A.B.V.); msanchez@ujaen.es (M.R.-S.); 2 North Jaén Area Health Authority, Andalusian Health Service (SAS), 23710 Jaén, Spain

**Keywords:** metabolic syndrome, glucagon-like peptide-1, dipeptidyl-peptidase 4, cardiovascular risk

## Abstract

Background: Glucagon-Like Peptide-1 (GLP-1) is hydrolyzed by Dipeptidyl-Peptidase 4 (DPP4), and several studies suggest that both GLP-1 and DPP4 inhibitors have potentially beneficial effects on cardiovascular risks. The objective of this study was to analyze the differences between plasma GLP-1 and DPP4 activity in male and female patients with metabolic syndrome, and its relationship with physiological and metabolic parameters. The study included 25 apparently healthy Controls (C) and 21 Metabolic Syndrome patients (MS). Anthropometric indices, cardiovascular risk-score, and Mediterranean Diet Adherence (AMeDit) were evaluated. Fasting glucose, glycosylated hemoglobin (HbA1c), and insulin were measured. Insulin, GLP-1, and plasma DPP4 were determined within the first 30-min postprandial period. Body-Mass-Index was significantly higher, and AMeDit was significantly lower, but only in MS women. However, fasting glucose, HbA1c, and postprandial insulin were significantly higher in MS men, but not in MS women. Postprandial GLP-1 levels were lower in C men than in C women. Interestingly, in comparison with controls, we found significant lower levels of plasma DPP4 in MS-women only. Moreover, negative lineal regressions were established between DPP4 activity with waist-to-hip ratio and cardiovascular risk-score, and positive lineal regression with AMeDit. These results indicate gender differences in the behavior of GLP-1 and DPP4 activity in MS, which could be relevant for its treatment with GLP-1 analogues and DPP4 inhibitors.

## 1. Introduction

Metabolic Syndrome (MS) is a common metabolic disorder that results from the increasing prevalence of obesity, hypertension, and type 2 diabetes in both developed and developing countries [1,2]. In addition, it is accompanied by a high risk of Cardiovascular Disease (CVD), and a pro-inflammatory state contributes to its development [3].

Several studies demonstrate a positive relationship between hyperglycemia and micro- and macrovascular complications [4], and this relationship spreads even into non-diabetic ranges of plasma glucose levels [5]. Although hyperglycemia is connected with an increasing risk of CVD, the glycemic control with diabetes medications has failed to reduce macro-vascular disease, demonstrating that this control alone is not sufficient to decrease CVD events. In this sense, a new class of hypoglycemic drugs, which raise or mimic glucagon-like peptide-1 (GLP-1), may also improve lipoprotein profiles, blood pressure control, weight loss, and endothelial function [6].

GLP-1 belongs to the incretin family of hormones and peptides able to modify the endocrine function of the pancreas and modulate insulin secretion, and is also able to inhibit gastric emptying, glucagon secretion, and food intake [7]. GLP-1 exerts its physiological actions by binding to a G-protein-coupled receptor (GLP-1R), expressed in pancreatic islet α and β cells and in peripheral tissues, including the central and peripheral nervous systems, heart, kidney, lung, and gastrointestinal tract [8].

GLP-1 secretion is stimulated in the distal small intestine and colon through the presence of carbohydrate [glucose] and others products of food digestion, such as free fatty acids, mainly oleic acid, being the L cell Fatty Acid Transport Proteins (FATPs) essential for oleic acid-induced GLP-1 release [9]. Indeed, several beneficial metabolic effects of the Mediterranean diet, rich in olive oil, are linked to high oleic acid ingestion [10], and the intake of a diet enriched in Monounsaturated Fatty Acids (MUFAs) increases plasma GLP-1 levels and improves glycemic control in diabetes type 2 and insulin-resistant patients [11].

GLP-1 is rapidly inactivated by Dipeptidyl-Peptidase-4 activity (DPP4, or T-cell activation antigen CD26 (EC 3.4.14.5.)), a serine exopeptidase that catalyzes the degradation of the two active circulating equipotent molecular forms [8,12].

DPP4 is a ubiquitous membrane-bound aminopeptidase, expressed in many tissues such as gut, liver, lung, kidney, and endothelial cells [13]. DPP4 preferentially cleaves peptides contain a proline or alanine residue in the second amino-terminal position, and the extracellular domain of DPP4 can also be cleaved from the membranes and circulates in plasma, where it keeps its enzymatic activity [8].

Dipeptidyl Peptidase 4 inhibitors (DPP4i) comprise the most widely used incretin-based therapy for the treatment of type 2 diabetes. These inhibitors prolong the half-life of incretins, such as GLP-1, and reduce modesty HbA1c levels. DPP4 is also expressed in blood vessels and myocardium, indicating an important role in cardiovascular regulation [14]. Moreover, circulating DPP4 is increased in obese and type 2 diabetic subjects, and it may be a link between obesity and vascular dysfunction [15]. Oral hypoglycemic agents that inhibit DPP4 activity, raise GLP-1 concentrations and increases the sensitivity of pancreatic cells to release insulin. 16-hydroxycleroda-3,13-dine-16,15-olide (HCD) is a natural supplement extracted from *Polyalthia longifolia* that exibits hypoglycemic activity. Encapsulation of HCD with Meso-porous Silica Nanoparticles (MSNs) resulted in a sustained release of HCD, an improved in the inhibition of DPP4, a better control of blood glucose, and a significantly decrease in the body weight of type 2 diabetic mice [16].

Previous studies have documented that circulating DPP4 originates from differentiated adipocytes and could be a novel adipokine that potentially links obesity to the metabolic syndrome [17]. Recent data also suggest that DPP4 is significantly associated with insulin resistance, others components of MS and several anthropometric parameters, such as Body Mass Index (BMI) and waist circumference [17,18]. However, although the link between DPP4 and various parameters related with MS has been extensively studied, no comprehensive studies have considered the sex influence in these relationships. Therefore, in this study we analyzed the gender differences between plasma GLP-1 and DPP4 activity in patients with metabolic syndrome and in their corresponding age-matched controls, as well as their relationship with some physiological and metabolic parameters.

## 2. Results

### 2.1. Anthropometric Indices, Cardiovascular Risk Score and Adherence to Mediterranean Diet

Significant gender differences were observed when both men and women controls were compared. Bicipital (*p* < 0.05) and tricipital (*p* < 0.001) skinfold were higher in women. Tricipital skinfold was also higher (*p* < 0.001) in MS women than in MS men. However, no differences between MS men and MS women were observed for bicipital skinfold. Waist-to-hip ratio was lower (*p* < 0.001) in MS women than in MS men, but no gender differences were observed between controls. Comparisons between MS groups with their corresponding controls demonstrated that body weight (*p* < 0.05) and BMI (*p* < 0.05) were significantly higher in MS-women, but not in MS-men. Several anthropometric indices such as waist circumference (*p* < 0.05 for men, *p* < 0.001 for women), waist-to-hip ratio (*p* < 0.01 for men and *p* < 0.001 for women), bicipital skinfolk (*p* < 0.05 for men and women), subscapular skinfold (*p* < 0.01 for men and women), and suprailiac skinfold (*p* < 0.01 for men and women) were increased in both MS groups. Cardiovascular risk score was elevated in MS men (*p* < 0.01) and women (*p* < 0.05), but AMeDit was significantly lower only in MS women (*p* < 0.05) (Table 1).

### 2.2. Biochemical Parameters

While plasma fasting glucose (*p* < 0.001), glycoxylated hemoglobin (HbA1c) (*p* < 0.01), postprandial insulin (*p* < 0.001) and GLP-1 (*p* < 0.05) were significantly higher in MS men than in control men, no differences were observed between MS and control women. Postprandial GLP-1 levels were higher in C women than in C men (*p* < 0.05). However, while fasting glucose did not differ between control men and women, the levels in MS women were significantly lower (*p* < 0.01) than in MS men (Table 2).

### 2.3. Dipeptidyl Peptidase 4 Activity

**Figure 1:**
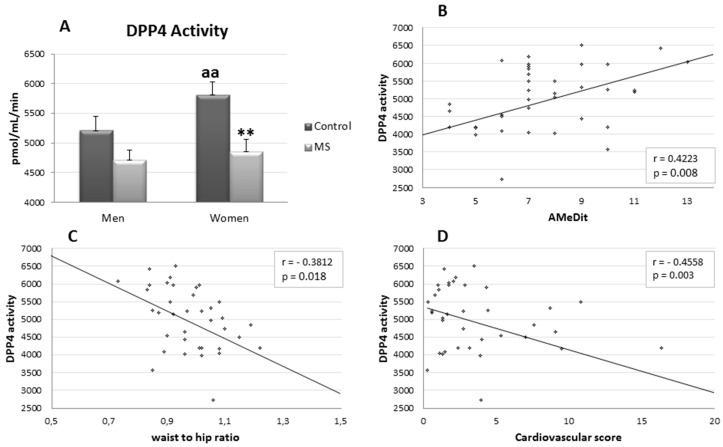
Graphic Panel (**A**) represents mean values ± standard error of postprandial plasma Dipeptidyl-Peptidase 4 activity (DPP4), expressed as pmol of l-Gly-Pro-4-Methoxy-β-naphtylamide hydrolyzed per minute of incubation and per mL of plasma, in the four groups studies: control men, control women, Metabolic Syndrome (MS) men and Metabolic Syndrome (MS) women. (*) indicates significant differences between metabolic syndrome v. control: ** *p* < 0.01. (a) indicates significant differences between women vs. men: aa *p* < 0.01. Graphics Panels (**B**–**D)** represent significant (*p* < 0.05) linear regressions established between DPP4 and AMeDit (**B**), waist-to-hip ratio (**C**) and cardiovascular score (**D**).

The postprandial plasma levels of DPP4 activity were higher in C women compared with C men (*p* < 0.01). However, no gender differences were observed in MS groups. Although DPP4 activity levels were lower in MS in comparison with controls, only in MS women was a statistical significance reached (*p* < 0.01) (Figure 1A). Moreover, negative linear regressions were established between DPP4 activity and waist-to-hip ratio (*p* < 0.05, r = −0.3812) (Figure 1C) , cardiovascular risk score (*p* < 0.01, r = −0.4558) (Figure 1D), and positive lineal regression with AMeDit (*p* < 0.01, r = 0.4223) (Figure 1B).

## 3. Discussion

The main objective of the present study was to evaluate the differences in GLP-1 levels and DPP4 activity between MS patients and control subjects, comparing men and women, together with the analysis of the relationship of these variables with anthropometric parameters.

In control groups, we found statistical differences in bicipital and tricipital skinfolds, with upper values in women compared with men. However, in MS groups the differences between men and women were established in tricipital skinfold and waist-to-hip ratio. Metabolic syndrome clusters increased several skinfolds (bicipital, tricipital, subscapular and suprailiac), waist circumference and waist-to-hip ratio in both men and women. Nevertheless, body weight and BMI were only significantly increased in the female group. These differences between male and female MS patients in anthropometric parameters have been previously reported [19]. Interestingly, BMI in control men and women were above 25, indicating overweight but not obesity associated with hypertension or insulin resistance, indicating that differences between groups were associated to metabolic conditions. 

Cardiovascular scores were higher in both MS groups, which is in agreement with the increase of cardiovascular risk in metabolic syndrome condition [20], but AMeDit was only lower in the MS women group, indicating the important role of dietary management in this group [21]. Therefore, our results support the existence of a differential development of MS between men and women, mainly associated with the regulation of body fat distribution and metabolic abnormalities by sex hormones [22].

With regard to the glycemic control, we found significant differences in fasting glucose and HbA1c only in MS men compared with his control, but not between women groups. Differences were not established among MS and control subjects in basal insulin. However, higher values of postprandial insulin (30 min after feeding) were measured in MS men than in control. These results indicate sex-related disturbances in glucose metabolism, although previous studies have reported a markedly higher prevalence of impaired glucose tolerance in women than in men, whereas the opposite was observed for impaired fasting glucose [23]. Nevertheless, the underlying mechanisms for these sex differences remain to be elucidated.

Although we do not find significant differences in postprandial levels of GLP-1 between men and women with MS, high levels of this peptide were observed in control women compared with control men. The peptide GLP-1 has been widely implicated in insulin secretion and glucose homeostasis. Beyond these effects, increasing evidence indicates that GLP-1 may exert several cardiovascular actions [7] and improves endothelial function in normal subjects and in patients with type 2 diabetes and coronary artery disease [24]. These cardiovascular protective actions could be especially important in MS, which is characterized by a high risk of cardiovascular disease. On the other hand, the presence of MS appears to modify the response to incretin-based therapies, and MS patients with elevated levels of GLP-1 are high-risk patients for cardiovascular disease, independent from the presence of diabetes [25].

The differences in GLP-1 between control men and women in our results could be related to the different pattern in the glycemic control, and a lower cardiovascular risk in women group. GLP-1 levels are reduced in patients with type 2 diabetes after a mixed meal or an oral glucose load [26,27]. However, we found an increase of GLP-1 release in MS men, probably associated to the size and compositions of meals with high oleic acid content [10,28].

It is more interesting to note the changes in DPP4 activity that were detected when we compared control men versus control women: the latter group had higher levels, which is in agreement with the higher levels of GLP-1. MS decreased DPP4 activity in both sexes, although only in women the difference is statistically significant, according to the higher levels of postprandial GLP-1 and DPP4 activity in control women. Previous results indicated that chronic exposure to hyperglycemia stimulates DPP4 expression and activity [29], but these results were obtained in subjects with >8.5% HbA1c. 

Furthermore, negative linear regressions were established between DPP4 activity with waist-to-hip ratio and cardiovascular risk score and a positive linear regression with AMeDit, indicating that the decrease in plasma DPP4 activity is related to higher cardiovascular risk. These results are interesting, because DPP4 activity has been proposed as a new adipokine [17], but glucose is able to inhibit adipocyte DPP4 release [30]. Clinical and experimental studies have demonstrated that DPP4 inhibitors are efficient in protecting cardiac and vascular systems through antiatherosclerotic and vasculoprotective mechanism. However, we find a negative significant regression between plasma DPP4 activity and cardiovascular risk scores, and significant higher levels of DPP4 activity and GLP-1 in control women, which is in agreement with a lower cardiovascular risk. However, in men, the MS does not significantly modify the DPP4 activity, but increases the levels of postprandial GLP-1. Several studies suggest that patients with type 2 diabetes, in general, do not exhibit reduced GLP-1 secretion in response to an Oral Glucose Tolerance Test (OGTT) or meal test [31]. Therefore, the different levels of this activity in men and women MS patients could be relevant to the treatment with GLP-1 analogues and DPP4 inhibitors.

## 4. Materials and Methods

### 4.1. Subjects

The study included 46 subjects, belonging to the North Jaén Area Health Authority, Andalusian Health Service (SAS) (Jaén, Spain). They were divided in two groups in a cross-sectional study: 25 healthy controls volunteers (C group, 44% men and 56% female) and 21 metabolic syndrome patients (MS group, 48% men and 52% female) (The Third Report National Cholesterol Education Program Expert Panel on Detection, Evaluation, and Treatment of High Blood Cholesterol in Adults, ATP III diagnostic criteria). The study protocol was in accordance with the Declaration of Helsinki and was approved by the Institutional Review Board of the North Jaén Area Health Authority. All participants were informed of the objectives of this study before they provided written consent to participate in it. The following exclusion criteria were considered: age under 50 and over 65 years old, post- menopausal women, abdominal hernia, an increase or decrease of 5 kg or more in body weight during last six months, type 1 diabetics, cardiac insufficiency, hepatic insufficiency or neoplastic disease, and treatment with insulin or incretin therapy.

### 4.2. Data Collection

Anthropometric measurements were made according to the recommendations of the International Standards for Anthropometric Assessment (ISAK, 2001). Body weight was measured using an electronic scale (±0.1 kg). BMI was calculated as weight divided by height squared (kg/m^2^). Criteria used to define overweight were the ones of the World Health Organization (WHO), as a BMI ≥30 kg/m^2^. Abdominal waist and hip circumferences were measured using a flexible steel tape (Holtain LTD, Crosswell, UK). Waist circumference was measured half-way between the lower costal border and the iliac crest. Hip circumference was measured at the level of the maximum extension of the buttocks posteriorly in a horizontal plane, without compressing the skin. Anthropometric skinfold considered were: tricipital (PT), bicipital (PBi), subscapular (PSb) and suprailiac (PS) and determined (mm) using a standard caliper (Holtain LTD).

Cardiovascular risks were calculated for each patient according to the criteria of the SCORE project [32]. The Mediterranean Diet Adherence (AMeDit) was estimated using a scale that indicates the degree of adherence to the traditional Mediterranean diet [33].

Plasma glucose and HbA1c were determined in fasting conditions (10 h) using an autosampler Hitachi (Roche) by glucose oxidase/peroxidase method and PNT ADAMS A1C HA 8160 for HbA1c measurement. A second blood sample was obtained in postprandial conditions 30 min after a breakfast (919.6 KJ) consisting of 44.2% carbohydrates, 35% lipids (26.6% saturated fatty acids, 61.1% monounsaturated fatty acids and 9.1% polysaturated fatty acids) and 20.8% protein. Insulin and GLP-1 levels were determined in these plasma samples with Ethylenediaminetetraacetic Acid (EDTA) and a DPP4 inhibitor (Millipore), using Bio-plex Pro™ magnetic bead-based assays (Bio-Rad Laboratories, Hercules, CA, USA) on the Bio-plex^®^ platform (Bio-Rad), according to the manufacturer’s instructions.

Dipeptidyl peptidase 4 activity levels were determined in plasma sample by a fluorimetric assay using l-Gly-Pro-4-Methoxy-β-naphthylamide as substrate. Briefly, 20 µL of each sample were incubated with 90 µL of a substrate solution at 37 °C. Fluorescence was measured continuously for 30 min. at 412 nm emission wavelength with an excitation wavelength of 345 nm in a FLUOstarOPTIMA microplate reader (BMG Labtech, San Diego, CA, USA). The fluorescence values obtained were converted to pmol of the released β-naphthylamine by extrapolating to a standard curve previously obtained after the determination of decreasing concentrations of β-naphthylamine in a medium equal to the corresponding substrate solution. Values were expressed as pmol of β-naphthylamine released per minute of incubation and per mL of plasma.

### 4.3. Statistical Analysis

Data were expressed as mean ± standard error. Statistical analyses were performed using the Statistical Package for the Social Sciences (SPSS 15.0, IBM, New York, NY, USA). One-way ANOVA was used in order to analyze mean differences among groups. When significant differences were found (*p* < 0.05), post hoc Tukey’s test was performed to identify the source of these differences. A generalized linear regression model was used to detect the interaction between DPP4 activity and anthropometrics, dietetic, or biochemical parameters.

## Figures and Tables

**Table 1:** Mean values ± standard error of body weight, Body Mass Index, anthropometrics skin folds (Bicipital, Tricipital, Subscapular and Suprailiac), waist circumference, waist-to-hip ratio, cardiovascular score, and Mediterranean diet adherence, corresponding to the four groups studied.

	Control Men	MS Men	Control Women	MS Women
Body weight (kg)	79.3 ± 4.06	90.1 ± 1.27	65.7 ± 2.87	80.6 ± 4.97 *
Body Mass Index (kg/m^2^)	27.8 ± 1.16	33.5 ± 1.66	26.7 ± 1.38	32.8 ± 1.95 *
Waist circunference (cm)	96.9 ± 3.68	112.9 ± 3.81 *	85.2 ± 2.48	106.1 ± 4.12 ***
Waist to hip ratio	1 ± 0.02	1.1 ± 0.03 **	0.9 ± 0.01	1 ± 0.01 *** aaa
Bicipital skinfold (mm)	7.4 ± 0.85	13.8 ± 1.55 *	13.1 ± 0.74 a	18.3 ± 2.09 *
Tricipital skinfold (mm)	10.9 ± 1.27	13.4 ± 1.56	23.1 ± 1.58 aaa	28.3 ± 2.65 aaa
Subscapular skinfold (mm)	18.2 ± 2.30	31.2 ± 1.98 **	20.2 ± 1.73	31.6 ± 2.64 **
Suprailiac skinfold (mm)	10.2 ± 1.07	19.9 ± 2.63 **	16.1 ± 1.43	23.8 ± 1.63 **
Cardiovascular score	2.3 ± 0.40	7.8 ± 1.67 **	1.2 ± 0.21	4.7 ± 1.47 *
AMeDit	8.7 ± 0.59	7.0 ± 0.67	8.6 ± 0.51	6.1 ± 0.58 *

AMeDit (Mediterranean diet adherence), MS men (Metabolic Syndrome men), MS women (Metabolic Syndrome women). (*) significant differences between metabolic syndrome vs. control: * *p* < 0.05, ** *p* < 0.01, *** *p* < 0.001. (a) significant differences between women vs. men: a *p* < 0.05, aaa *p* < 0.001.

**Table 2:** Mean values ± standard error of fasting plasma glucose, fasting insulin, glycosylated hemoglobin, postprandial insulin, and plasma GLP-1 levels in the four groups studied.

	Control Men	MS Men	Control Women	MS Women
Fasting glucose (mg/dL)	96.4 ± 3.60	174.1 ± 17.99 ***	90.4 ± 2.14	115.1 ± 9.96 aa
Fasting insulin (µU/mL)	7.8 ± 1.15	11.6 ± 2.76	5.6 ± 0.68	10.9 ± 1.34
HbA1 (%)c	5.4 ± 0.12	7.9 ± 0.58 **	5.5 ± 0.07	6.5 ± 0.53
Postprandial insulin (µU/mL)	9.5 ± 1.09	17.5 ± 2.66 ***	13.0 ± 1.27	17.2 ± 2.35
GLP-1 (pg/mL)	34.7 ± 4.20	53.6 ± 8.28	68 ± 9.66 a	52.9 ± 6.55

HbA1 (glycoxylated hemoglobin), GLP-1 (glucagon-like peptide 1), MS (metabolic sysndrome). (*) significant differences between metabolic syndrome v. control: ** *p* < 0.01, *** *p* < 0.001. (a) significant differences between women v. men: a *p* < 0.05, aa *p* < 0.01.
